# Human uterine and placental arteries exhibit tissue-specific acute responses to 17β-estradiol and estrogen-receptor-specific agonists

**DOI:** 10.1093/molehr/gat095

**Published:** 2013-12-19

**Authors:** Jemma J. Corcoran, Christopher Nicholson, Michèle Sweeney, Jayne C. Charnock, Stephen C. Robson, Melissa Westwood, Michael J. Taggart

**Affiliations:** 1 Maternal and Fetal Health Research Centre, Institute of Human Development, University of Manchester, Manchester Academic Health Sciences Centre, St Marys Hospital, Manchester M13 9WL, UK; 2 Institute of Cellular Medicine, 3rd Floor Leech Building, Medical School, Faculty of Medical Sciences, Newcastle University, Framlington Place, Newcastle upon Tyne NE2 4HH, UK

**Keywords:** estrogen, ER agonists, human uterine arteries, human placental arteries, vascular tone

## Abstract

The discrete regulation of vascular tone in the human uterine and placental circulations is a key determinant of appropriate uteroplacental blood perfusion and pregnancy success. Humoral factors such as estrogen, which increases in the placenta and maternal circulation throughout human pregnancy, may regulate these vascular beds as studies of animal arteries have shown that 17β-estradiol, or agonists of estrogen receptors (ER), can exert acute vasodilatory actions. The aim of this study was to compare how acute exposure to ER-specific agonists, and 17β-estradiol, altered human placental and uterine arterial tone *in vitro*. Uterine and placental arteries were isolated from biopsies obtained from women with uncomplicated pregnancy delivering a singleton infant at term. Vessels were mounted on a wire myograph, exposed to the thromboxane receptor agonist U46619 (10^−6^ M), and then incubated with incremental doses (5 min, 0.03–30 µM) of either 17β-estradiol or agonists specific for the ERs ERα (PPT), ERβ (DPN) or the G-protein-coupled estrogen receptor GPER-1 (G1). ERα and ERβ mRNA expression was assessed. 17β-estradiol, PPT and DPN each relaxed myometrial arteries (*P* < 0.05) in a manner that was partly endothelium-dependent. In contrast, 17β-estradiol or DPN relaxed placental arteries (maximum relaxation to 42 ± 1.1 or 47.6 ± 6.53% of preconstriction, respectively) to a lesser extent than myometrial arteries (to 0.03 ± 0.03 or 8.0 ± 1.0%) and in an endothelial-independent manner whereas PPT was without effect. G1 exposure did not inhibit the constriction of myometrial nor placenta arteries. mRNA expression of ERα and ERβ was greater in myometrial arteries than placental arteries. ER-specific agonists, and 17β-estradiol, differentially modulate the tone of uterine versus placental arteries highlighting that estrogen may regulate human uteroplacental blood flow in a tissue-specific manner.

## Introduction

A key determinant of appropriate fetal growth during human pregnancy is the transfer across the placental epithelium of oxygen and nutrients between the uterine and fetal circulations. As the two circulations are separate physical entities—blood from the maternal uterine circulation that traverses through the lumen of terminal open-ended spiral arterioles is directed towards the syncytial surface of the placenta but does not come in to direct contact with the enclosed fetoplacental circulatory flow—then materno-fetal nutrient exchange will be influenced by changes in either the uterine or placental vasculatures. This makes it important to understand how the blood vessels from each circulation respond to physiological vasoactive factors. The placenta lacks autonomic innervations (Myatt, 1992), which suggests that factors within the local environment may be crucial for this purpose.

Estrogen may be one such modulator of uteroplacental vascular function. Estrogen's effect on vascular tone has been documented in many experimental and clinical contexts but not in relation to human pregnancy. Estrogen action via two receptors, ERα (also known as ESR1) and ERβ (also known as ESR2), classically initiates transcriptional changes (Miller and Duckles, 2008). However, acute vasodilatory actions of estrogen have pointed to non-genomic actions of ERα and ERβ. These may be mediated via endothelial-dependent and/or -independent mechanisms (Shaw *et al.*, 2000; Hisamoto and Bender, 2005) with the former involving regulation of nitric oxide (NO) bioavailability. Interestingly, a transmembrane G-protein-coupled receptor, GPER-1 (also known as GPR30), has also been identified as an ER capable of mediating rapid non-genomic signalling (Revankar and Prossnitz, 2005; Feldman and Gros, 2011) and it is of interest to unravel whether estrogenic vasoactive actions may be mediated, in part or wholly, via activation of this receptor.

In sheep *in vivo*, the uterine circulation exhibits a rapid vasodilatory response to 17β-estradiol administration (Pastore *et al.*, 2012). Such actions may in part reflect activation of endothelial NO synthase (NOS; Chen *et al.*, 2004) and in rat uterine arteries isolated from non-pregnant rats, but not those from late pregnant rats, 17β-estradiol-mediated relaxations were partly blunted by NOS inhibition (Scott *et al.*, 2007). The involvement of ERs in these actions is a matter of considerable debate. Certainly, reports of variations in the relative abundance of mRNA encoding ERα or ERβ in uterine endothelial cells (Critchley *et al.*, 2001; Liao *et al.*, 2005; Pastore *et al.*, 2012) points to the possibility of receptor-specific estrogenic responses in the uterine circulation. In comparison to these data, however, it is striking that there is little information about the estrogenic effects on placental blood vessels. The placenta is an important source of estrogen, evinced by the substantial increase in maternal plasma level (You *et al.*, 2006) and the fetal adrenal gland contributes to estrogen within the placental circulation (Smith *et al.*, 2009). Thus, locally derived and/or locally acting estrogen may modulate the vasoreactivity of the human uterine and placental circulations. Of note, it has been suggested that the endothelium of human fetoplacental blood vessels express ERβ protein but that ERα is absent or expressed at much less levels (Su *et al.*, 2007, 2009).

The availability of agonists specific for ERα (PPT: (4,4′,4″-(4-propyl-[^1^H]-pyrazole-1,3,5-triyl) tris-phenol)), ERβ (DPN: (2,3-bis(4-hydroxyphenol)-propionitrile)), or GPER-1 (G1) has enabled mapping of the non-genomic vasodilatory actions resulting from stimulation of the different ERs across many vascular beds (Montgomery *et al.*, 2003; Hass *et al.*, 2009; Feldman and Gros, 2011). However, a direct comparison of the acute estrogenic influences on arteries isolated from two human vasculatures, and from patients with no medical complications, has not been examined.

Therefore, we have investigated the effect of 17β-estradiol and ER-specific agonists on the tone of human placental and uterine (myometrial) arteries isolated from biopsies of normal pregnant women with no underlying pathophysiology. Our data point to a new understanding of human uteroplacental vascular regulation by reporting that estrogen regulates the tone of arteries isolated from these circulations in ER- and tissue-specific manners. This also alerts us to the possibility that variations in estrogenic responsiveness may exist across other human arterial tissues.

## Materials and Methods

### Chemicals and solutions

ER agonists were purchased from R&D systems (UK); other chemicals, unless stated, were from Sigma (UK) or VWR (UK).

### Sample collection and tissue microdissection

The study was approved by Local Research Ethics Committees and tissue collected following written, informed consent from women with uncomplicated, singleton term pregnancies (38–42 weeks gestation). Arteries were dissected as described previously (Corcoran *et al.*, 2012). Chorionic plate arteries (diameter 284 ± 18 µm) were dissected from placental biopsies obtained within 30 min of vaginal or elective Caesarean (not in labour) delivery and placed directly into ice-cold tissue collection buffer ((TCB); modified Krebs solution −154 mM NaCl/5.4 mM KCl/1.2 mM MgSO_4_ · 7H_2_O/10 mM MOPS/5.5 mM glucose/1.6 mM CaCl_2_ · 2H_2_O; pH 7.4). Myometrial arteries (diameter 209 ± 23 µm) were obtained from biopsies (∼1 cm^3^) taken at the time of Caesarean section from the upper segment of the uterine incision and placed into ice-cold TCB.

### Myography experiments and analysis

Four segments from each artery were mounted onto a wire myograph (610M; Danish Myotechnologies, Denmark). After a 20 min equilibration period, the arteries were stretched by 100 μm and allowed to equilibrate for 2 min and tension was recorded using a Myodaq software package (Danish Myotechnologies, Denmark). The vessels were stretched a further 100 μm and the procedure was repeated in at least three steps until the passive tension exceeded 5.1 kPa for human placental arteries [vessel tension expressed as active wall tension (Δ*T* in mN/mm) can be transformed to active effective pressure (Δ*T*/(diameter/2000)) denoted by kPa] or 13.3 kPa for human myometrial arteries. Arteries were then normalized to a resting diameter equivalent to 0.9 of L_5.1 kPa_ (placental) or L_13.3 kPa_ (myometrial) as described previously (Corcoran *et al.*, 2012), and equilibrated in physiological salt solution (PSS: 127 mM NaCl, 4.7 mM KCl, 2.4 mM MgSO_4_ · 7H_2_O, 25 mM NaHCO_3_, 1.18 mM KH_2_PO_4_, 0.07 mM EDTA, 1.6 mM CaCl_2_ · 2H_2_O, 6.05 mM glucose at pH 7.4) at 37°C. Myometrial arteries were bubbled with a 95%air–5%CO_2_ mixture and placental arteries with 5% O_2_–5% CO_2_–90% N_2_ (Corcoran *et al.*, 2012).

Arteries were preconstricted with 1 μM of the thromboxane receptor agonist U46619 (9,11-dideoxy-9a,11a-methanoepoxy prostaglandin F_2a_, Merck Millipore, UK) and vasoactive actions of estrogenic compounds examined by adding incremental doses (5 min duration each, of 0.05, 0.15, 0.3, 0.6, 1, 2, 5, 10, 30 µM) of either 17β-estradiol, PPT, DPN, G1 or vehicle (ethanol). The range of doses was chosen to be akin to that reported in studies on other vessel types (Shaw *et al.*, 2000; Montgomery *et al.*, 2003; Hisamoto and Bender, 2005; Scott *et al.*, 2007). In some experiments, the role of the endothelium was investigated by abrading the arterial lumen to render the endothelium dysfunctional or preincubating with the NOS inhibitor L-NNA (10 µM). Arteries were subsequently constricted with 1 µM U46619 and cumulative dose–response curves to estrogenic compounds assessed. Endothelial functional integrity, determined by the relaxant actions of 1 µM bradykinin on U46619 preconstrictions in myometrial arteries, was 81 ± 10% before and 19 ± 12% after abrasion. In another set of experiments, placental arteries were preincubated with the L-type Ca^2+^ channel agonist Bay K8644 (1,4-dihydro-2,6-dimethyl-5-nitro-4-(2-[trifluoromethyl]phenyl)pyridine-3-carboxylic acid methyl ester), further constricted with U46619 and the effects of 17β-estradiol or DPN examined.

In a subset of experiments, arteries were treated with α-toxin so as to render the plasmalemma permeable to small solutes and ions and enable direct investigation of the Ca^2+^-sensitivity of contraction by directly controlling the level of activating Ca^2+^ surrounding the myofilaments. The procedure followed was as reported previously (Wareing *et al.*, 2005; Dordea *et al.*, 2013). In brief, intact PSS-equilibrated vessels were exposed to a mock intracellular solution (G1: sodium creatine phosphate 10 mM; Na_2_ATP 5.2 mM; magnesium methanesulphonate 7.3 mM; potassium methane sulphonate, 74 mM; K_2_EGTA 1 mM buffered to pH 7.1 with 30 mM of the chemical buffer PIPES and addition of KOH) for 15 min and then treated with a 25 μl droplet of permeabilizing cocktail [pCa 6.7 solution (see below) supplemented with 500 U/ml α-toxin plus 10 μmol/l A23187] for 30 min. The cocktail was removed by washing the vessels with G1 solution followed by equilibration of the permeabilized arteries in pCa9 (relaxing) solution ([Ca^2+^]_i_ = 0.001 μM; sodium creatine phosphate, 10 mM; Na_2_ATP, 5.2 mM; magnesium methane sulfonate, 7.3 mM, potassium methane sulfonate, 74 mM; K_2_EGTA 1 mM; PIPES, 30 mM; pH 7.1 with KOH). Permeabilization was assessed by exposing vessels to a high calcium activating solution (pCa4.5 ([Ca^2+^]_i_ = 31.5 μM)) followed by relaxation with pCa9 solution. Vessels were exposed to a sub-maximal calcium activating solution (pCa6.7 ([Ca^2+^]_i_ = 0.2 μM)) and thence U46619 (1 μM) in the continued presence of pCa6.7 solution. Any additional constriction was thus due to sensitization of the contractile filaments to the sub-maximal activating Ca^2+^ by the presence of U46619. The experiment was completed by exposure to a maximal dose (30 μM) of either 17-β estradiol or DPN to examine any effect of these drugs on agonist-dependent Ca^2+^-sensitization of contraction.

Data recorded (Myodaq; Danish Myotechnologies, Denmark) as active wall tension (Δ*T* in mN/mm) was transformed to active effective pressure (Δ*T*/(diameter/2000)) denoted by kPa. Data were Arcsine transformed to enable statistical analysis of dose–response curves by two-way repeated-measures analysis of variance with Bonferroni *post hoc* testing (significance taken as *P* < 0.05) and presented graphically as mean ± SEM. *E*_max_ (maximal effect) and pIC50 (−Log(IC50)) of drugs were calculated and effects of endothelial abrasion and NOS inhibition analysed using *t*-test.

### Quantitative polymerase chain reaction

Vessels were homogenized in lysis buffer and total RNA extracted as described (Dordea *et al.*, 2013). 100 ng RNA from each sample was reverse transcribed using AffinityScript cDNA synthesis kit (Stratagene, USA) including a DNase treatment step to eliminate genomic DNA contamination. ERα and ERβ were amplified using specific primers (ERα nm_000125: forward—gct gct ggc tac atc; reverse—agg act cgg tgg ata tgg; ERβ nm_001040275: forward—ata ctt tcc tcc tat gta gac; reverse—tgt gat aac tgg cga tgg) and quantified using a Stratagene MX3000P machine and Stratagene Brilliant SYBR Green I QPCR mastermix. cDNA from human myometrial artery and placental artery samples were assessed on the same 96-well plate and for each gene of interest data from each sample were expressed relative to the internal reference cDNA sample [reverse transcribed from human reference total RNA (Stratagene Human Reference Total RNA, La Jolla, USA)] also assessed on every 96-well plate and analysed using an independent *t*-test; *P* < 0.05 was considered significant.

### Western blotting

Arteries were homogenized in (1 mg per 5 µl) 50 mM Tris–HCl, 150 mM NaCl, 1% NP-40, 0.5% Na-deoxycholate, 1 mM EGTA (pH7.4) with 2% (vol/vol) protease inhibitor and 0.5% (vol/vol) phosphatase inhibitor (Sigma) and prepared for western blotting as described by Dordea *et al.* (2013). Twenty to 30 µg protein from each sample was resolved by SDS–PAGE and transferred to PVDF membranes for western blotting with antiserum against ERβ (Abcam #ab288 mouse monoclonal) or ERα (Santa Cruz Biotechnology #H-184 rabbit polyclonal 1:1000; Santa Cruz Biotechnology #MC-20 rabbit polyclonal 1:1000; Vector Labs #VP613 mouse monoclonal, 1:1000) and membranes visualized chemiluminescently following probing with a HRP-goat anti-mouse-IgG (1:5000; DakoCytomation, #PO447) or HRP-goat anti-rabbit-IgG (1:5000; DakoCytomation, #PO448) antibody. The optical densities from the western blot scans were determined using Intelligent Quantifier Software (BioImage Systems Inc.). Protein expression was quantified by comparing the optical density of the arterial band of interest to the optical density of the positive control following the subtraction of background signal. PVDF membranes were stained with naphthol blue black in order to visualize actin expression and assess for equal protein loading between lanes.

## Results

### 17β-estradiol and ER agonists have differing relaxant actions on human myometrial and placental arteries

**Figure 1:**
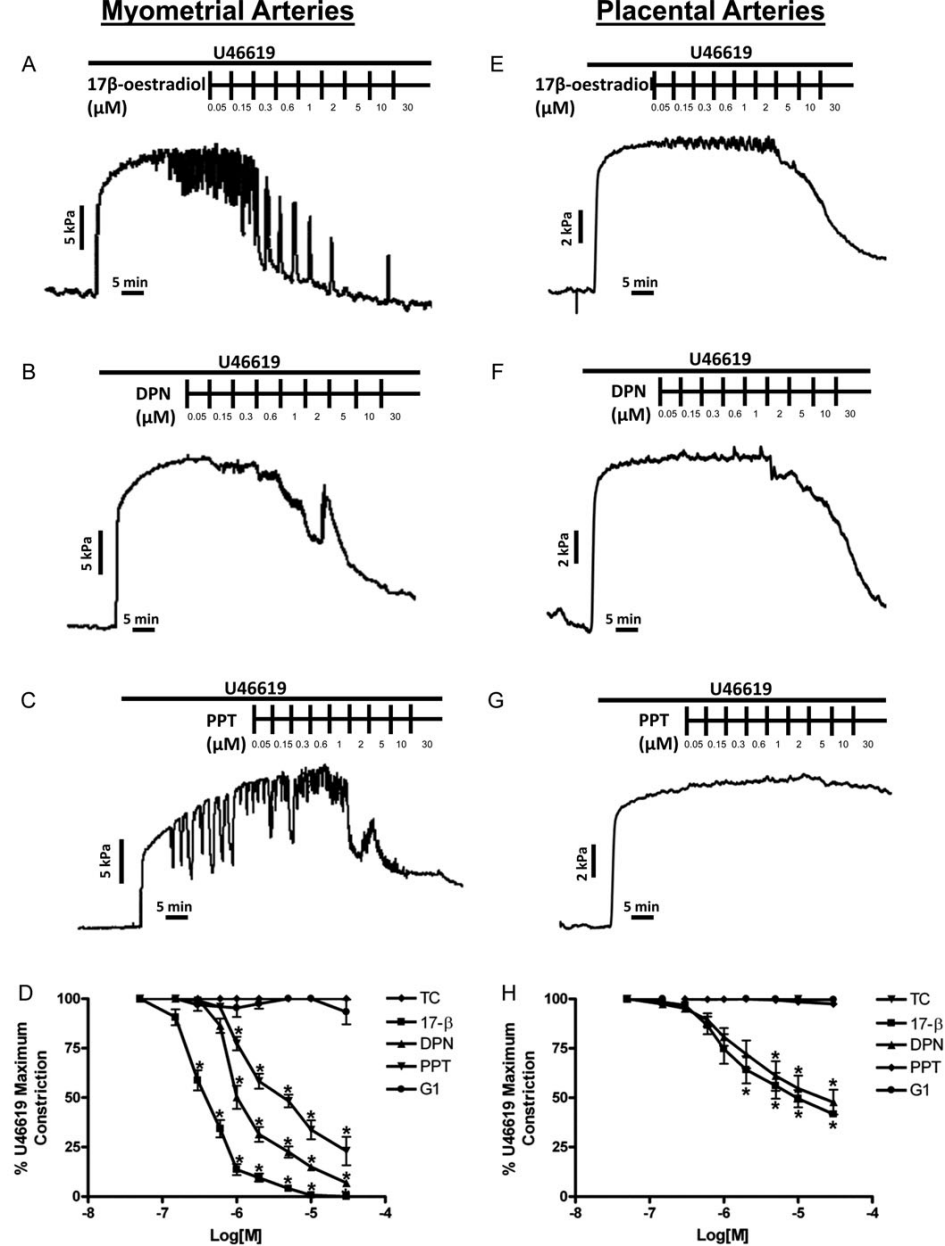
Differential effects of 17β-estradiol and ER agonists on human myometrial and placental arteries. Myometrial (**A**–**D**) or placental (**E**–**H**) arteries were preconstricted with U46619 and exposed to incremental doses of estrogenic compounds. Representative raw tracings are displayed in A–C, E–G, summary data (mean ± SEM, *n* = 6) in D and H. 17β-estradiol or ERβ agonist, DPN relaxed myometrial arteries to a greater extent than placental arteries whereas ERα agonist, PPT relaxed only myometrial arteries. GPER-1 agonist (G1) was without effect in either artery type. *Significant differences from time control (TC).

17β-estradiol induced significant vasodilation of preconstricted human myometrial arteries from 0.3 to 30 μM (relaxations to 59 ± 5.1%–0.03 ± 0.03% of preconstriction, Fig. 1A and D). DPN also induced acute vasodilation from 1 to 30 μM (relaxations to 87 ± 3.1%–8.0 ± 1.0% of preconstriction, Fig. 1B and D) and PPT from 2 to 30 μM (relaxations to 77 ± 3.3%–37 ± 3.9% of preconstriction, Fig. 1C and D). In contrast, human placental arteries (Fig. 1E–H) exhibited less vasodilation to 17β-estradiol (from 1 to 30 μM: 76 ± 7.1%–42 ± 1.1% of preconstriction; *P* < 0.001) or DPN (from 2 to 30 μM: 72 ± 7.05%–47.6 ± 6.53% preconstriction; *P* < 0.006) than myometrial arteries and showed no relaxation to PPT (Fig. 1G and H). G1 had no relaxant actions in myometrial or placental arteries (Fig. 1D and H).

### Acute relaxatory effects are partly endothelial- and NO-mediated in human myometrial arteries but are endothelial-independent in human placental arteries

**Figure 2:**
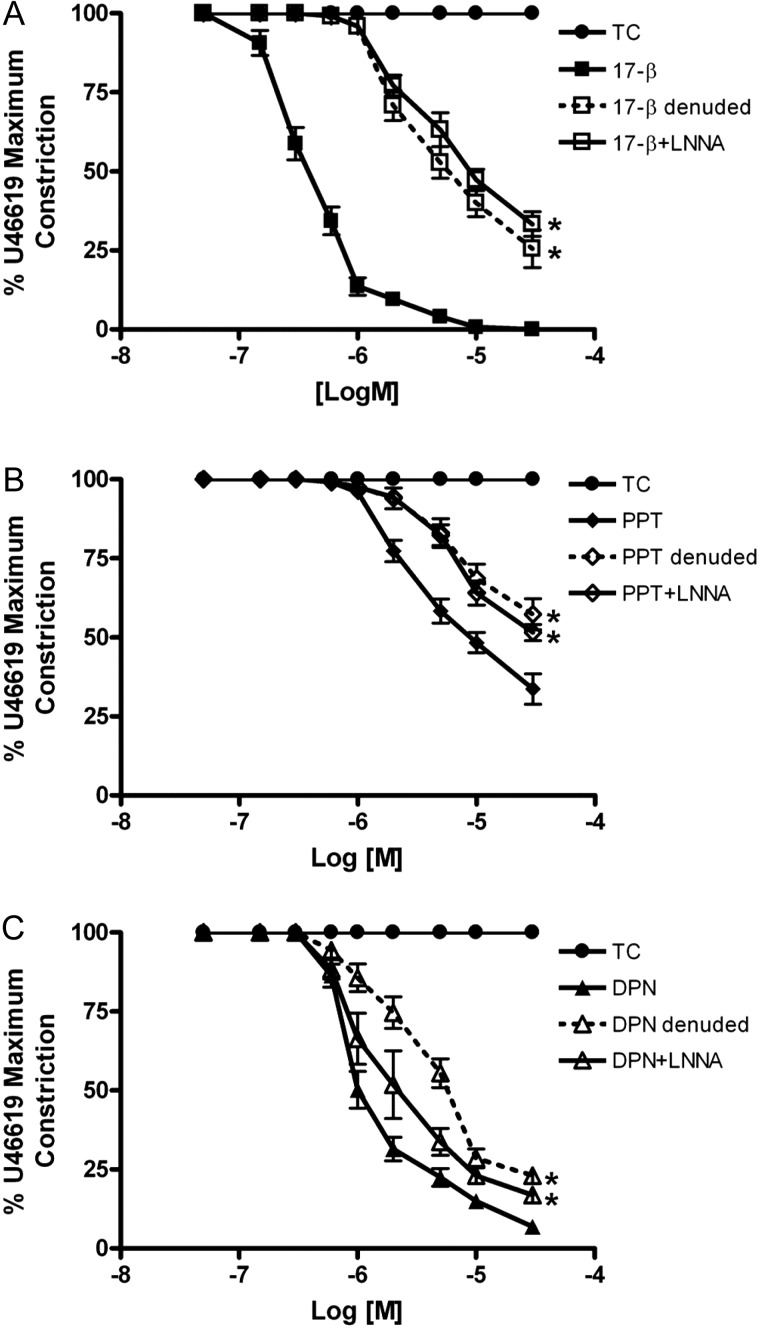
Relaxatory actions of estrogenic compounds are partly endothelial- and NO-dependent in human myometrial arteries. The relaxation of preconstricted myometrial arteries by 17β-estradiol (**A**), PPT (**B**) or DPN (**C**) was blunted following endothelial abrasion (open symbols, dashed lines) or by pretreatment with nitric oxide synthase inhibitor (L-NNA) (open symbols, solid lines). *Significant differences from intact artery responses. Data are presented as mean ± SEM (*n* = 6).

The vasodilatory effects of 17β-estradiol, DPN or PPT on human myometrial arteries were each blunted following endothelial abrasion (Fig. 2A–C). In endothelial abraded vessels, the relaxation to 17β-oestradiol was 71 ± 4.8%–26 ± 1.8% (0.3–30 µM), to DPN was 49 ± 10%–17 ± 2.3% (2–30 µM) and to PPT was 83 ± 4.5%–57 ± 4.9% (5–30 µM) of preconstriction. Similar results were obtained in endothelial-intact arteries exposed to L-NNA (Fig. 2A–C).

The data in Table I shows that either treatment—endothelial abrasion or preincubation with L-NNA—in myometrial arteries significantly reduced the maximal relaxation (*E*_max_) induced by each agonist and mostly resulted in a reduction in sensitivity (pIC50) to each agent.

**Table I:** Effects of endothelial abrasion or NOS inhibition on the estrogenic relaxatory responses of human myometrial arteries.

	17β-estradiol	PPT	DPN
*E*_max_			
Intact	0.03 ± 0.03	33.7 ± 4.8	6.9 ± 1.7
Denuded	25.5 ± 5.9^a^	57.3 ± 4.9^a^	23.0 ± 2.4^a^
+LNNA	33.3 ± 3.9^a^	51.5 ± 2.6^a^	16.8 ± 2.3^a^
pIC50			
Intact	6.43 ± 0.04	5.42 ± 0.06	5.99 ± 0.04
Denuded	5.47 ± 0.10^a^	5.17 ± 0.07^a^	5.48 ± 0.10^a^
+LNNA	5.34 ± 0.13^a^	5.14 ± 0.07^a^	5.84 ± 0.11

The maximum (*E*_max_) and half-maximal (pIC50) relaxation values of preconstricted myometrial arteries by 17β-estradiol, DPN or PPT.

^a^Significant difference from paired intact artery (*t*-test). Data are presented as mean ± SEM (*n* = 6).

**Figure 3:**
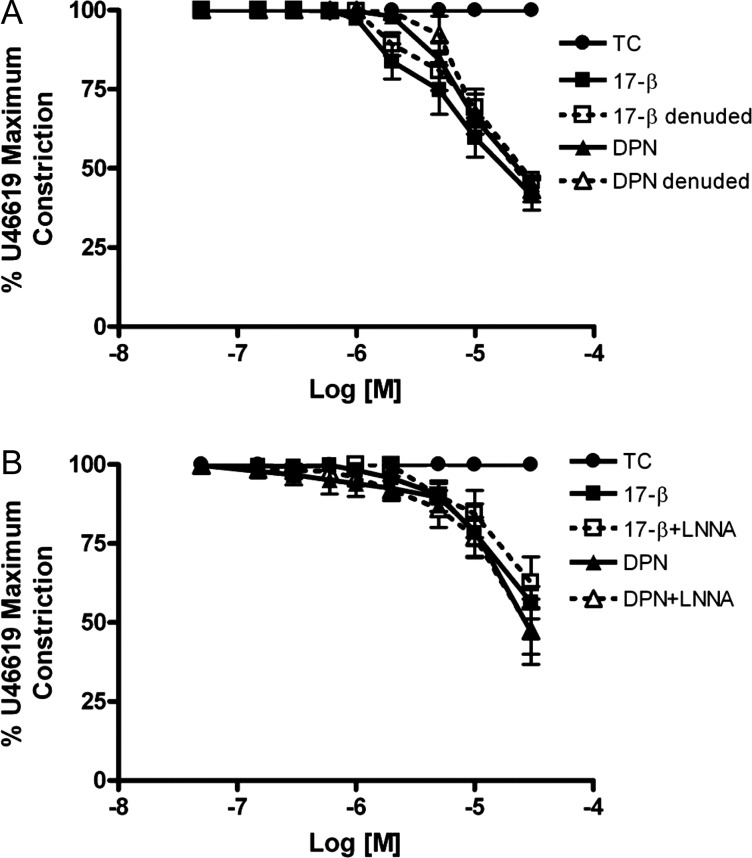
Vasodilatory actions of estrogenic compounds are endothelial-independent in human placental arteries. The relaxation of preconstricted placental arteries by 17β-estradiol or DPN were unaltered by endothelial abrasion (**A**) or by pretreatment with L-NNA (**B**). Data are presented as mean ± SEM (*n* = 6).

In contrast to the effects in intact human myometrial arteries, the vasodilatory actions of 17β-estradiol or DPN on human placental arteries were unaffected by endothelial abrasion (Fig. 3A) or by exposure of endothelial-intact arteries to L-NNA (Fig. 3B).

### Differential expression of ERα and ERβ between human myometrial and placental and arteries

**Figure 4:**
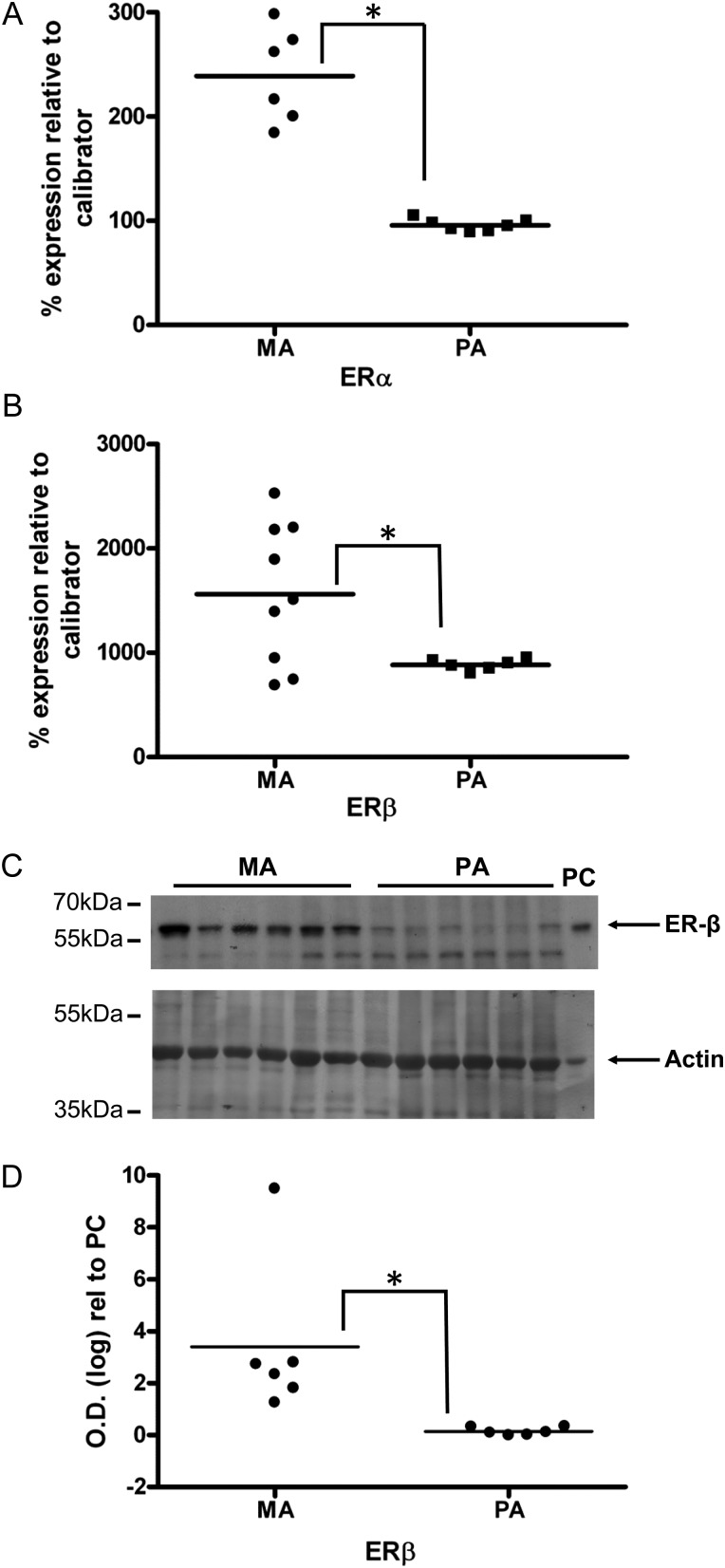
ER expression in human arteries. Expression of mRNA encoding for ERα (**A**) or ERβ (**B**). ERα mRNA expression was greater in myometrial arteries (MA) than placental arteries (PA). Western blotting indicated that ER expression was greater in myometrial than placental arteries (**C** and **D**). PC, refers to the positive control of human myometrium. *Significant differences between MA and PA.

Analysis of ER mRNA demonstrated that both types of artery express ERα (Fig. 4A) and ERβ (Fig. 4B). In myometrial arteries the level of mRNA encoding ERα (*P* < 0.01) and ERβ (*P* < 0.04) was greater than placental arteries. The Ct values of ERα in the myometrial and placental arteries were, respectively, 24.35 ± 0.11 (*n* = 6) and 25.43 ± 0.21 (*n* = 6) (corresponding calibrator RNA 25.43 ± 0.11). The Ct values of ERβ in the myometrial and placental arteries were, respectively, 25.54 ± 0.34 and 25.32 ± 0.02 (corresponding calibrator RNA 28.67 ± 0.15). Western blotting also showed that ERβ protein was greater in myometrial than placental arteries (Fig. 4C and D). However, in spite of using three separate anti-ERα antibodies (detailed in the Materials and Methods) we were unable to consistently visualize staining that could be specifically attributable to ERα in myometrial or placental arteries for the purposes of quantitative comparisons.

### Vasodilatory effects of estrogen do not involve altered Ca^2+^-sensitization of contraction but are enhanced in the presence of an L-type Ca^2+^ channel agonist

**Figure 5:**
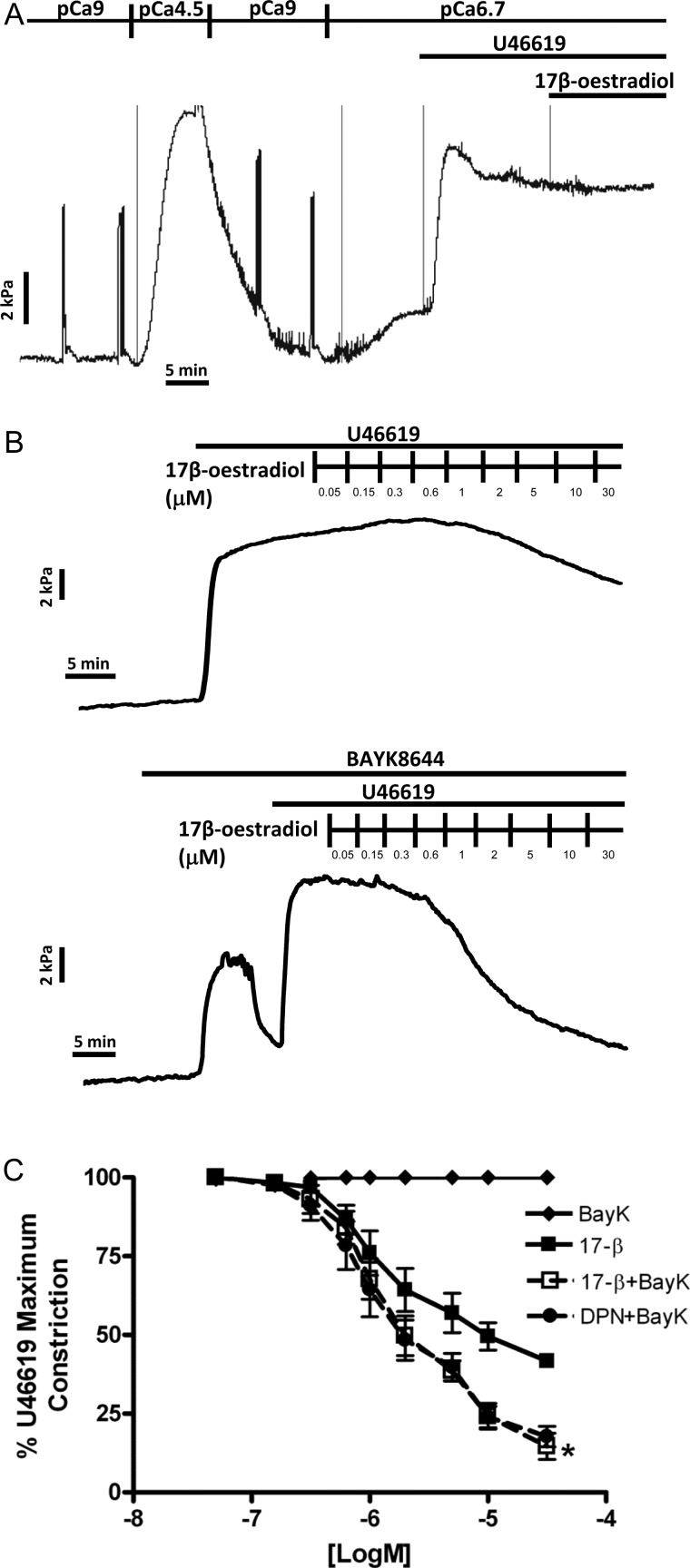
Vasodilatory effects of estrogenic compounds are unrelated to changes in Ca^2+^-sensitization of contraction but are enhanced in the presence of an L-type Ca^2+^ channel agonist. Permeabilized placental arteries exhibited U46619-induced Ca^2+^-sensitization of contraction at sub-maximal activating Ca^2+^ (pCa6.7) that was similar in magnitude to contraction in pCa4.5 solution alone (representative raw data tracing of **A**). 17β-estradiol (30 µM) did not affect this agonist-mediated Ca^2+^-sensitization. In intact preconstricted placental arteries, 17β-estradiol-mediated relaxation (**B**) was enhanced by preincubation with the L-type Ca^2+^ channel agonist Bay K8644 (**C** and **D**). Similar effects were observed on DPN-mediated relaxation (D). *Significant differences between 17β-estradiol alone and in the presence of Bay K8644.

Figure 5A indicates a representative raw tracing of an α-toxin permeabilized placental artery exhibiting U46619-induced Ca^2+^-sensitization of contraction at sub-maximal activating Ca^2+^ (pCa6.7). This was of similar magnitude to the preceding contraction induced by maximally activating pCa4.5 solution. Addition of 30 µM 17β-estradiol was without effect on this agonist-mediated Ca^2+^-sensitization (*n* = 4). In intact preconstricted placental arteries, the relaxation by 17β-estradiol (B) was enhanced by preincubation with the L-type Ca^2+^ channel agonist Bay K8644 (C and D). Similar effects were observed on DPN-mediated relaxation (D).

## Discussion

Efficient uteroplacental blood perfusion is required for a successful pregnancy outcome yet the factors and mechanisms involved in controlling vascular tone within the human uterus and placenta are poorly understood. This study is the first to investigate the potential role of estrogen and estrogen receptors in acutely regulating the vasoreactivity of arteries isolated from these circulations at the end of pregnancy. Our results demonstrate that the relaxatory influences of 17β-estradiol or ER-specific agonists differ between myometrial and placental arteries. This suggests that estrogen regulation of human uteroplacental vascular tone occurs in a tissue-specific manner and opens the possibility that similar variations may occur between other human vascular beds.

17β-estradiol acutely relaxed preconstricted human myometrial arteries. The ERβ-specific agonist DPN and ERα-specific agonist PPT also exerted vasodilatory effects. The GPER-1 agonist G1 was without effect. These data point to estrogenic vasodilation of human myometrial arteries via ERβ and ERα. In our study, endothelial abrasion, or NOS inhibition, resulted in a blunting of relaxatory responses to all three agents thus indicating that estrogen relaxation of myometrial arteries is mediated via endothelial-dependent and -independent mechanisms. In sheep, uterine artery endothelial cells express ERβ and ERα and several estrogenic influences occur in an ER-specific manner (Byers *et al.*, 2005; Pastore *et al.*, 2012). ERβ and ERα have previously been suggested to mediate non-genomic stimulation of NO production (Wu *et al.*, 2011) and this may underlie the endothelial-dependent relaxant action of estrogen.

Human placental arteries relaxed to a lesser extent to 17β-estradiol or DPN than myometrial arteries. However, there was no relaxation to PPT or G1. Furthermore, the influence of 17β-estradiol or DPN in placental arteries was unaffected by endothelial abrasion or NOS inhibition. This implicates a prominent role for ERβ, but not ERα or GPER-1, in acute endothelial-independent estrogenic relaxation of human placental arteries and illustrates profound differences to human myometrial arteries. The lack of endothelial-dependence in estrogen action in placental arteries may be related to a relatively weak stimulation of NO-mediated relaxation by endothelial-dependent agonists in this vessel type (Sweeney *et al.*, 2008) but is unlikely to be related to impaired sensitivity of placental artery smooth muscle cells to NO production (Hayward *et al.*, 2013). In addition, in sheep uterine arterial endothelial cells the relative expression of endothelial NOS is greater than that of placental arteries (Byers *et al.*, 2005). The endothelial-independent mechanisms of actions of estrogen in other vessels have been suggested to arise via inhibition of voltage-gated Ca^2+^ entry to smooth muscle cells (Shaw *et al.*, 2000; Montgomery *et al.*, 2003). Although in human myometrial and placental arteries this requires further elucidation we have identified two important facets of the possible smooth muscle-related mechanisms of estrogenic action in placental arteries. First, the relaxations are unlikely to involve the modulation of intracellular pathways affecting myofilament Ca^2+^-sensitization. Secondly, that voltage-gated Ca^2+^ entry is likely to be a target of acute estrogenic actions because, in the presence of an L-type Ca^2+^ channel activator, which one may anticipate to have facilitated plasmalemmal Ca^2+^ entry because contractility is increased, the vasodilatory estrogenic influences are actually enhanced. Further such work, in alliance with approaches such as siRNA-mediated receptor knockdown, or the application of ER-specific antagonists, is needed to establish whether the tissue-specific responses we observe are due entirely to ER expression patterns (see below) or the tissue-specific activation of other post-receptor intracellular signalling modalities. This has recently been suggested, for example, to be important in these human tissues' distinct responses to IGF-I or protein kinase G stimulation (Corcoran *et al.*, 2012; Dordea *et al.*, 2013).

The lack of vasodilatory influence of the GPER-1 agonist G1 on either artery type was surprising given reports indicating a relaxant action on human internal mammary artery tone (Hass *et al.*, 2009). This may reflect tissue-specific actions of G1; however, we investigated the acute effects (within 5 min of each dose) of G1 whereas the vasodilatory influence on mammary arteries was observed over a longer time-course (Hass *et al.*, 2009).

Maternal estradiol levels rise continually throughout gestation with peak values reported to be ∼60 nM (Norman *et al.*, 1989; O’ Leary *et al.*, 1991; Smith *et al.*, 2009). Assessments of estradiol in the uterine or placental circulations have suggested greater values than in the systemic maternal circulation with peaks of ∼350–500 nM (Smith *et al.*, 1979). Notwithstanding the possibility that the concentration of estradiol at the cellular site of action may be greater than that measured in the circulation(s), these *in vivo* circulating estradiol levels are within the lower-to-mid-range of our dose–response curves designed to investigate the acute influences on preconstricted tone. A caveat to our study, of course, is that *in vivo* the human uterine or placental arteries will be chronically, rather than acutely, exposed to estrogens.

The differential ER-mediated responses in human myometrial and placental arteries supports a number of animal studies suggesting ER- and tissue-specific estrogenic vasodilatory actions (Montgomery *et al.*, 2003; Al Zubair *et al.*, 2005; Bolego *et al.*, 2005; Ma *et al.*, 2010; Patker *et al.*, 2011). Herein, we found that mRNA encoding ERα and ERβ are present in both artery types. The expression of ERβ mRNA or protein was significantly greater in human myometrial than placental arteries. In addition, ERα mRNA was greater in myometrial than placental arteries. Of note, the vasodilatory influence of DPN was less in placental arteries than myometrial vessels and PPT was an ineffective relaxant in placental arteries. ERβ has been suggested to be the prominent ER expressed in endothelial cells of stem villous placental arteries and endometrial uterine vessels (Critchley *et al.*, 2001; Su *et al.*, 2007). These arteries are both downstream of the resistance-like arteries used in the present study and points, therefore, to a need to consider if there are functionally relevant ER expression differences across the human uterine or placental vascular trees. The unreliability (in our hands) of a number of anti-ERα antibodies to give definitive identification by western blotting, coupled to the small amount of vascular material available for extensive optimization procedures, has hampered our ability to fully examine the ER tissue- and cell-specific protein expression patterns. Nonetheless, the lack of endothelial-dependence of DPN- or 17β-estradiol-mediated relaxation of placental arteries suggests a predominating influence of ERβ-related events at the level of placental smooth muscle cells where, indeed, ERβ appears to be expressed (Su *et al.*, 2011). In uterine arteries, on the other hand, the vasodilations to 17β-estradiol, DPN or PPT involved activation of signalling pathways in both endothelial and smooth muscle cells.

The present study is important for identifying (i) that estrogen differentially regulates human uterine and placental vascular tone; (ii) the potential of ER-specific agonists to act as tissue-specific modulators of human uteroplacental vascular tone; (iii) adding to an evolving narrative in the literature whereby circulatory, or locally produced, factors may differentially alter the tone of human myometrial or placental arteries (Hemmings *et al.*, 2006; Hudson *et al.*, 2007; Corcoran *et al.*, 2012). It is of interest that subcutaneous arteries isolated from post-menopausal healthy women relaxed more to PPT than 17β-estradiol and the effects of either agent were unaltered by NOS inhibition (Cruz *et al.*, 2008)—results that were different to that which we observed in maternal myometrial arteries. These data, therefore, also alerts us to the possibility that, more generally, estrogenic modulation of human arterial tone may occur in a tissue-specific manner and thereby contribute to distributing blood flow in an organ-specific fashion.

## Authors' roles

J.J.C., C.N., M.S. and J.C.C. performed the experiments and analysed the data. S.C.R. facilitated human tissue provision. M.W. and M.J.T. conceived and designed the study and prepared the manuscript. All authors approved the final version.

## Funding

This work was supported by Tommy's: the Baby Charity; the Medical Research Council; and the NIHR Newcastle Biomedical Research Centre. The Manchester Maternal and Fetal Health Research Centre is supported by funding from the Greater Manchester Local Comprehensive Research Network. Funding to pay the Open Access publication charges for this article was provided by the University of Manchester.

## Conflict of interest

None declared.

## References

[GAT095C1] Al Zubair K, Razak A, Bexis S, Docherty JR (2005). Relaxations to oestrogen receptor subtype selective agonists in rat and mouse arteries. Eur J Pharmacol.

[GAT095C2] Bolego C, Cignarella A, Sanvito P, Pelosi V, Pellegatta F, Puglisis L, Pinna C (2005). The acute estrogenic dilation of rat aorta is mediated solely by selective estrogen receptor-α agonists and is abolished by estrogen deprivation. J Pharmacol Exp Ther.

[GAT095C3] Byers MJ, Zangl A, Phernetton TM, Lopez G, Chen D-b, Magness RR (2005). Endothelial vasodilator production by ovine uterine and systemic arteries: ovarian steroid and pregnancy control of ERα and ERβ levels. J Physiol.

[GAT095C4] Chen DB, Bird IM, Zheng J, Magness RR (2004). Membrane estrogen receptor-dependent extracellular signal-regulated kinase pathway mediates acute activation of endothelial nitric oxide synthase by estrogen in uterine artery endothelial cells. Endocrinology.

[GAT095C5] Corcoran JJ, Charnock JC, Martin J, Taggart MJ, Westwood M (2012). Differential effect of insulin like growth factor-I on constriction of human uterine and placental arteries. J Clin Endocrinol Metab.

[GAT095C6] Critchley HO, Brenner RM, Henderson TA, Williams K, Nayak NR, SLayden OD, Millar MR, Saunders PT (2001). Estrogen receptor beta, but not estrogen receptor alpha, is present in the vascular endothelium of the human and nonhuman primate endometrium. J Clin Endocrinol Metab.

[GAT095C7] Cruz MN, Agewall S, Schenck-Gustafsson K, Kublickiene K (2008). Acute dilatation to phytoestrogens and estrogen receptor subtypes expression in small arteries from women with coronary heart disease. Atherosclerosis.

[GAT095C8] Dordea AC, Sweeney M, Taggart J, Lartey J, Wessel H, Robson SC, Taggart MJ (2013). Differential vasodilation of human placental and myometrial arteries related to myofilament Ca2+-desensitization and the expression of Hsp20 but not MYPT1. Mol Hum Reprod.

[GAT095C9] Feldman RD, Gros R (2011). Unravelling the mechanisms underlying the rapid vascular effects of steroids: sorting out the receptors and the pathways. Br J Pharmacol.

[GAT095C10] Hass E, Bhattacharya I, Brailoiu E, Damjanovic M, Brailou GC, Gao X, Mueller-Guerre L, Marjon NA, Gut A, Minotti R (2009). Regulatory role of G protein-coupled estrogen receptor for vascular function and obesity. Circ Res.

[GAT095C11] Hayward CE, Higgins L, Cowley EJ, Greenwood SL, Mills TA, Sibley CP, Wareing M (2013). Chorionic plate arterial function is altered in maternal obesity. Placenta.

[GAT095C12] Hemmings DG, Hudson NK, Halliday D, O'Hara M, Baker PN, Davidge ST, Taggart MJ (2006). Sphingosine-1-phosphate acts via rho-associated kinase and nitric oxide to regulate human placental vascular tone. Biol Reprod.

[GAT095C13] Hisamoto K, Bender JR (2005). Vascular cell signaling by membrane estrogen receptors. Steroids.

[GAT095C14] Hudson NK, O'Hara M, Lacey HA, Corcoran J, Hemmings DG, Wareing M, Baker PN, Taggart MJ (2007). Modulation of human arterial tone during pregnancy: the effect of the bioactive metabolite Sphingosine-1-Phosphate. Biol Reprod.

[GAT095C15] Liao WX, Magness RR, Chen D (2005). Expression of estrogen receptors-α and -β in the pregnant ovine uterine artery endothelial cells in vivo and in vitro. Biol Reprod.

[GAT095C16] Ma Y, Qiao X, Falone AE, Reslan OM, Sheppard SJ, Khalil RA (2010). Gender-specific reduction in contraction is associated with increased estrogen receptor expression in single vascular smooth muscle cells of female rat. Cell Physiol Biochem.

[GAT095C17] Miller VM, Duckles SP (2008). Vascular actons of estrogens: functional implications. Pharmacol Rev.

[GAT095C18] Montgomery S, Shaw L, Pantelides N, Taggart MJ, Austin C (2003). The acute effects of oestrogen receptor subtype-specific agonists on vascular contractility. Br J Pharmacol.

[GAT095C19] Myatt L (1992). Control of vascular resistance in the human placenta. Placenta.

[GAT095C20] Norman M, Eriksson CG, Eneroth P (1989). A comparison between the composition of maternal peripheral plasma and plasma collected from the retroplacental compartment at caesarean section. A study on protein and steroid hormones and binding proteins. Arch Gynecol Obstet..

[GAT095C21] O'Leary P, Boyne P, Flett P, Beilby J, James I (1991). Longitudinal assessment of changes in reproductive hormones during normal pregnancy. Clin Chem.

[GAT095C22] Pastore MB, Jobe SO, Ramadoss J, Magness RR (2012). Estrogen receptor-α and estrogen receptor-β in the uterine vascular endothelium during pregnancy: functional implications for regulating uterine blood flow. Semin Reprod Med.

[GAT095C23] Patker S, Farr TD, Cooper E, Dowell FJ, Carswell HVO (2011). Differential vasoactive effects of oestrogen, oestrogen receptor agonists and selective oestrogen receptor modulators in rat middle cerebral artery. Neurosci Res.

[GAT095C24] Revankar CB, Prossnitz ER (2005). A transmembrane intracellular estrogen receptor mediates rapid cell signalling. Science.

[GAT095C25] Scott PA, Tremblay A, Brochu M, St-Louis J (2007). Vasorelaxant action of 17-estradiol in rat uterine arteries: role of nitric oxide synthases and estrogen receptors. Am J Physiol.

[GAT095C26] Shaw L, Taggart MJ, Austin C (2000). Mechanisms of 17α-oestradiol-induced vasodilataion in isolated pressurised rat small arteries. Br J Pharmacol.

[GAT095C27] Smith R, Klopper A, Hughes G, Wilson G (1979). The compartmental distribution of oestrogens and pregnancy specific beta1 glycoprotein. Br J Obstet Gynaecol..

[GAT095C28] Smith R, Smith JI, Shen X, Engel PJ, Bowman ME, McGrath SA, Bisits AM, McElduff P, Giles WR, Smith DW (2009). Patterns of plasma corticotrophin-releasing hormone, progesterone, estradiol and estriol change and the onset of human labor. J Clin Endocrinol Metab.

[GAT095C29] Su EJ, Cheng YH, Chatterton RT, Lin ZH, Yin P, Reierstad S, Innes J, Bulun SE (2007). Regulation of 17-β hydroxysteroid dehydrogenase type 2 in human placental endothelial cells. Biol Reprod.

[GAT095C30] Su EJ, Lin ZH, Zeine R, Yin P, Reierstad S, Innes JE, Bulun SE (2009). Estrogen receptor-beta mediates cyclooxygenase-2 expression and vascular prostanoid levels in human placental villous endothelial cells. Am J Obstet Gynecol.

[GAT095C31] Su EJ, Ernst L, Abdallah N, Chatterton R, Xin H, Monsivais D, Coon J, Bulun SE (2011). Estrogen receptor-β and fetoplacental endothelial prostanoid biosynthesis: a link to clinically demonstrated fetal growth restriction. J Clin Endocrinol Metab.

[GAT095C32] Sweeney M, Wareing M, Mills TA, Baker PN, Taggart MJ (2008). Characterisation of tone oscillations in placental and myometrial arteries from normal pregnancies and those complicated by pre-eclampsia and growth restriction. Placenta.

[GAT095C33] Wareing M, O'Hara M, Seghier F, Baker PN, Taggart MJ (2005). The involvement of Rho-associated kinases in agonist-dependent contractions of human maternal and placental arteries at term gestation. Am J Obstet Gynecol.

[GAT095C34] Wu Q, Chambliss K, Umetani M, Mineo C, Shaul PW (2011). Non-nuclear estrogen receptor signalling in the endothelium. J Biol Chem.

[GAT095C35] You X, Yang R, Tang X, Gao L, Ni X (2006). Corticotrophin-releasing hormone stimulates estrogen biosynthesis in cultured human placental trophoblasts. Biol Reprod.

